# Association between fat mass index and fat-free mass index values and cardiovascular risk in adolescents

**DOI:** 10.1016/j.rppede.2015.06.020

**Published:** 2016

**Authors:** Patrícia Morais de Oliveira, Fabiana Almeida da Silva, Renata Maria Souza Oliveira, Larissa Loures Mendes, Michele Pereira, Ana Paula Carlos Cândido

**Affiliations:** Universidade Federal de Juiz de Fora (UFJF), Juiz de Fora, MG, Brazil

**Keywords:** Cardiovascular diseases/risk factors, Adolescent/growth and development, Body composition, Body fat

## Abstract

**Objective:**

To describe the association between fat mass index and fat-free mass index values and factors associated with cardiovascular risk in adolescents in the city of Juiz de Fora, Minas Gerais.

**Methods:**

Cross-sectional study was with 403 adolescents aged 10–14 years, from public and private schools. Anthropometric, clinical, and biochemical measurements were obtained, as well as self-reported time spent performing physical exercises, sedentary activities and sexual maturation stage.

**Results:**

Regarding the nutritional status, 66.5% of the adolescents had normal weight, 19.9% were overweight and 10.2% were obese. For both genders, the fat mass index was higher in adolescents who had high serum triglycerides, body mass index and waist circumference.

**Conclusions:**

Adolescents who had anthropometric, clinical and biochemical characteristics considered to be at risk for the development of cardiovascular disease had higher values of fat mass index. Different methodologies for the assessment of body composition make health promotion and disease prevention more effective.

## Introduction

The increased prevalence of overweight in adolescents^1^ is associated with risk factors for cardiovascular disease in adulthood and results in high costs for public health.^1^ Currently, it is shown that body fat distribution has more influence than total body mass in the presence of cardiovascular risk factors.^2^^–^ 4 The ability to measure or quantify body fat stores is central for preventing and treating obesity-related diseases.^2^ Therefore, more accurate methods are needed to assess adiposity and do proper screening for early intervention.^3^^–^ 6

Body mass index (BMI) has been the most widely used anthropometric method for diagnosing overweight, but its predictive ability to identify young people with high body fat is open for discussion in scientific circles.^5^^,^ 6 It cannot discriminate between fat mass and lean mass, does not reflect the great changes in body composition that occur in this age group and are different between the sexes.^5^ So it would be more feasible to distinguish the body components through more precise measurements that consider the body fat percentage.^2^

VanItallie et al.^7^ proposed the use of fat mass index (FMI) and fat-free mass index (FFMI) for a more detailed anthropometric measurement, according to body compartments, by calculation that considers the amount of fat mass and fat-free mass in kg obtained by bioelectrical impedance, with the advantage of relating only one body weight component to the height squared and of being expressed in units that are common to BMI.^8^ With the use of these two indices, it becomes possible to judge whether a deficit or excess of body weight is selectively due to a change in fat-free mass, in fat mass, or both.^8^ Four typical situations can be identified: low FFMI and high FMI, corresponding to obesity; low FFMI and low FMI, corresponding to leanness; high FFMI and low FMI, corresponding to muscle hypertrophy; and high FFMI and high FMI, corresponding to combined excess of FFMI and FMI. The reference values of these two indices are not yet a consensus in the scientific literature, particularly for adolescents. In the study by Nakao and Komiya,^9^ the reference values of FFMI were 12.7–13.4kg/m^2^ for boys and 12–13kg/m^2^ for girls. As for FMI, the adopted reference values adopted were 2.8–3.6kg/m^2^ for boys and 3.2–3.8kg/m^2^ for girls.

Considering the need for a more detailed anthropometric assessment for adolescents, the aim of this study was to describe the relationship between the values of fat mass index and fat-free mass index and factors associated with cardiovascular risk in adolescents in the Juiz de Fora city, MG.

## Method

Cross-sectional study was performed with adolescents, aged 10–14 years, attending public and private schools in Juiz de Fora, MG. The number of schools and students per institution, belonging to that age group, was obtained through the School Census provided by INEP 2009.^10^ Thirty-five schools were selected, based on the proportion by city regions. Total sample was based on three parameters: proportion of obesity in the age group studied (8%)^11^; a desired precision of 2% was accepted, with a significance level of 5%; and 20% losses were considered due to the absence of adolescents in days of data collection or refusal to participate (no consent of teen, or parent/guardian). Students were randomly selected through the table of random numbers and stratified according to sex, age, and proportion in each school.

Anthropometric, clinical, and biochemical measurements were evaluated. Students also reported how much time they spent practicing exercises, watching TV, playing video games and using the computer. Adolescents who engaged in over 300 minutes per week of physical activity were considered active.^12^ Tanner's scale^13^ was used for adolescents’ self-assessment of their sexual maturation, and the girls indicated the presence and age of menarche occurrence.

Body mass was measured using a bipolar bioimpedance scale (Tanita^®^BC-553, Illinois, USA) with a maximum capacity of 136kg, also used to assess body fat percentage. Height was measured by field stadiometer (Alturaexata^®^, Belo Horizonte, Brazil), with scale in centimeters and accuracy of 0.01m. The nutritional status of the adolescents was determined by BMI for age, according to the WHO classification criteria, where a BMI above the 85th percentile is classified as overweight.^14^ Waist circumference was obtained using a 1.5m simple and inelastic tape measure, with 0.01m interval. All measurements followed the procedures standardized by the WHO, and circumference values above the 85th percentile were considered high for both sexes. Regarding fat percentage, values greater than 30% for girls and 25% for boys were considered high and of risk.^15^

Tetrapolar bioimpedance Biodynamics 310^®^ (Biodynamics Corporation, Washington, USA) was used with measurements performed according to the manufacturer's instructions. The resistance and reactance values found were used to determine the amount in kg of fat mass (FM) and fat-free mass (FFM). FMI was determined by dividing fat mass (in kilograms) by height squared (in meters). Similarly, FFMI was calculated by dividing lean mass (in kilograms) by height squared (in meters), as proposed by VanItallie et al.^7^ The average of the two closest values of the biceps, triceps, subscapular, and supra-iliac skinfold thickness measurements were added. Triceps and subscapular mean values of skin folds were also added separately. The analog skinfold caliper Lange^®^ (Beta Technology, California, USA), with precision of 0.1mm in triplicate, was used to measure the skinfold of biceps (performed with bent elbows at 90°, halfway between the armpit and the cubital fossa), triceps (at midpoint of the distance between the acromion and the olecranon, on the back of the left arm), subscapular (in the lower angle of the scapula, at 45° diagonal), and supra-iliac (on the mid-axillary line, between the last rib and the iliac crest). The formula proposed by Deurenberg et al.^16^ was used for data analysis to predict values associated with body density and subsequently those associated with fat in relation to body weight.

Blood samples (6mL) were collected by venipuncture in the antecubital region of patients after 12-h fast to measure the levels of total cholesterol, fractions (HDL-c and LDL-c), and triglycerides. The procedures were standardized in the Biochemistry Laboratory of the School Hospital of the Federal University of Juiz de Fora (UFJF). To evaluate lipid changes, the reference values recommended by the Brazilian Society of Cardiology were applied.^17^ Values above 150mg/dL, 100mg/dL, and 100mg/dL for total cholesterol, triglycerides, and LDL-c, respectively, were considered high risk for adolescents of both sexes; whereas for HDL-c, values below 45mg/dL were considered risk for both boys and girls. Diagnosis of diabetes mellitus was determined according to the Brazilian Society of Diabetes criteria (fasting glucose above 100mg/dL).^18^ The parameters were analyzed by automatic analyzer Cobas Mira Plus^®^ (Roche Diagnostics, Switzerland).

Blood pressure levels of students undergoing evaluation were measured three times with digital oscillometric device Omron HEM-705CP^®^ (Omron Corporation, Brazil). The subjects were seated (left arm extended at heart level), and an interval of 10–15min was maintained between each measurement. Classification was based on the 5th Brazilian Guidelines on Hypertension from the average of the three measurements performed (systolic blood pressure and diastolic blood pressure above the 90th percentile).^19^

The project was approved by the Federal University of Juiz de Fora Institutional Review Board, Opinion No. 09/2010, and started after the consent of the legal guardians of adolescents and school directors.

In the statistical analysis, Student's *t*-test was used to assess the differences between the mean values of each variable in relation to sex. The analysis of variance (ANOVA) was used to compare mean values of body composition indices and its corresponding masses with the other studied variables. The SPSS version 17.0 software was used for statistical analysis, and a 5% significance level was admitted.

## Results

A total of 403 adolescents were evaluated, 218 (54.1%) were female, mean age was 12.4±1.2 years. Age group distribution was similar between the sexes (*p*=0.30). The sample consisted of 66.5% eutrophic, 19.9% overweight, and 10.2% obese adolescents. Table 1 shows the characteristics of the study population stratified by sex.

**Table 1:** Comparison of the mean values of demographic, anthropometric, clinical, and biochemical variables and physical activity of the study population by sex (n=403). Juiz de Fora, MG, Brazil, 2014.

Variable	Girls (n=218)			Boys (n=185)		*p-* value a
	n	Mean±SD		n	Mean±SD	
Age (years)	218	12.4±1.1		185	12.3±1.1	0.30
Height (cm)	217	155.5±8.5		185	155.8±10.4	0.74
Weight (kg)	217	49.3±13.2		185	48.0±12.8	0.33
BMI (kg/m^2^)	218	20.2±4.6		185	19.5±3.5	0.16
Bipolar body fat (%)	209	25.3±7.8		175	17.5±7.7	<0.01
Waist circumference (cm)	213	66.9±9.9		183	67.7±9.7	0.39
Sum of 4 folds (mm)	197	60.7±26.1		170	50.1±26.0	<0.01
Sum of triceps and subscapular folds (mm)	197	31.5±13.1		170	26.7±13.1	<0.01
Fat-free mass (kg)	218	33.9±8.3		185	35±12.4	0.30
Fat-free mass index	218	13.9±2.9		185	14.2±4.1	0.42
Fat mass (kg)	218	13.9±8.8		185	10.3±6.4	<0.01
Fat mass index	218	5.7±3.4		185	4.8±2.5	<0.01
Mean systolic blood pressure (mmHg)	218	109.0±9.9		184	110.3±10.8	0.22
Mean diastolic blood pressure (mmHg)	218	68.1±6.5		184	66.5±7.0	<0.05
Physical activity time (min/week)	213	171.4±120.8		181	269.1±165.7	<0.01
Time watching TV, video game, and computer (h/week)	213	8.0±4.5		183	10.6±5.4	<0.01
Total cholesterol (mg/dL)	202	153.1±26.2		176	150.4±25.9	0.32
Glucose (mg/dL)	201	82.2±7.9		173	82.4±9.6	0.88
Triglycerides (mg/dL)	202	69.6±30.7		176	67.2±32.3	0.47
High density lipoprotein cholesterol (mg/dL)	162	48.7±8.9		145	46.7±11.2	0.09
Low density lipoprotein cholesterol (mg/dL)	162	90.9±23.3		145	90.8±20.5	0.95

a Student's *t* -test.

Regarding body composition, female adolescents had higher percentages of body fat (determined by bipolar bioimpedance and sum of skinfolds), FM and FMI. Mean diastolic blood pressure was higher in females. Male adolescents spent more time on physical activity and also in sedentary practices, such as watching TV, playing video games, and using the computer, than female adolescents.

Tables 2 and 3 represent the mean value and standard deviation of anthropometric, clinical, and biochemical characteristics in relation to body composition indices and its corresponding masses for both female and male adolescents, respectively. For both sexes, higher mean values of FFM, FFMI, FM and FMI were found in adolescents with excess weight, high body fat and waist circumference. There was a significant difference in systolic blood pressure. Girls with SAP>p90 had higher mean values of FM and FMI, and boys in the same PAS classification had higher mean values of FM and FFM. Regarding sexual maturation, FFMI and FFM were higher in the postpubertal stage for both girls and boys. The same was seen for FM and FMI in female adolescents in postpubertal stage. In biochemical evaluation, female adolescents with HDL-c<45mg/dL had higher mean FFM, FFMI, FM and FMI; whereas triglyceride values were higher in female adolescents with high FM and FMI.

**Table 2:** Comparison between anthropometric, clinical, biochemical and physical activity characteristics regarding fat-free mass, fat-free mass index, fat mass and fat mass index of female adolescents. Juiz de Fora, MG, Brazil, 2014.

Variables		n	Fat-free mass Mean±SD	Fat-free mass index Mean±SD	Fat mass Mean±SD	Fat mass index Mean±SD
*Body mass index*						
	<P85	155	32.1±7.3 *	13.4±2.6 *	10.0±4.4 *	4.1±1.6 *
	>P85	62	38.7±7.7 *	15.5±2.4 *	24.0±8.9 *	9.6±3.3^a^ *

*Bipolar fat*						
	<30%	154	32.2±7.3 *	13.4±2.6 *	10.2±4.4 *	4.2±1.7 *
	>30%	54	39.2±8.0 *	15.6±2.6 *	25.0±9.0 *	9.9±3.4 *

*Waist circumference*						
	<P 85	165	32.3±7.2 *	13.5±2.5 *	10.6±4.8 *	4.4±1.9 *
	>P 85	48	39.3±8.2 *	15.5±2.8 *	25.3±9.6 *	10.0±3.6 *

*Systolic blood pressure*						
	<P90	198	33.7±7.6	13.9±2.5	13.2±7.7 *	5.4±2.99 *
	>P90	19	35.7±13.9	13.9±5.2	21.8±13.9 *	8.5±5.4 *

*Diastolic blood pressure*						
	<P90	202	34.1±7.8	14.0±2.6	13.9±8.2	5.6±3.1
	>P90	15	31.1±13.3	12.9±5.3	15.1±14.4	6.2±5.7

*Physical activity*						
	>300min/week	21	35.0±6.3	14.8±1.6	15.2±9.4	6.3±3.6
	<300min/weeka	192	33.7±8.5	13.8±3.0	13.8±8.7	5.6±3.3

*Sexual maturation*						
	Prepubertal	54	28.6±8.1*^a^	13.0±3.3*^a^	9.5±6.5*^a^	4.2±2.7*^a^
	Pubertal	150	35.4±6.7*^b^	14.2±2.4*^b^	14.8±7.6*^b^	5.9±3.0*^b^
	Postpubertal	7	43.9±7.4*^c^	16.2±1.9*^b^	31.7±17.2*^c^	11.8±6.7*^c^

*Total cholesterol*						
	<150mg/dL	94	34.8±8.9	13.9±3.2	14.0±8.1	5.6±3.1
	>150mg/dL	108	33.5±8.0	13.9±2.7	14.4±9.4	5.8±3.6

*Glucose*						
	<100mg/dL	198	34.0±8.5	13.9±3.0	14.1±8.8	5.7±3.4
	>100mg/dL	3	34.1±4.5	13.7±0.8	14.8±10.1	5.8±3.9

*Triglycerides*						
	<100mg/dL	171	33.7±8.6	13.8±3.1	13.5±8.0 **	5.4±3.0 **
	>100mg/dL	31	36.2±7.2	14.8±1.8	17.7±12.1 **	7.2±4.7 **

*High density lipoprotein cholesterol*						
	>45mg/dL	111	32.7±8.4 *	13.4±3.1 *	12.9±8.1 *	5.2±3.1 *
	<45mg/dL	51	37.0±8.3 *	14.8±2.8 *	18.1±10.0 *	7.2±3.9 *

*Low density lipoprotein cholesterol*						
	<100mg/dL	104	34.4±9.2	13.8±3.4	14.2±8.0	5.6±3.0
	>100mg/dL	58	33.4±7.3	14.1±2.4	15.3±10.7	6.3±4.1

Means with different letters are significantly different according to the Least Significant Difference test (LSD).Student's *t* -test.

* *p* <0.01;.

** *p* <0.05.

**Table 3:** Comparison between anthropometric, clinical, biochemical and physical activity regarding fat-free mass, fat-free mass index, fat mass and fat mass index of male adolescents. Juiz de Fora, MG, Brazil, 2014.

Variables		n	Fat-free mass Mean±SD	Fat-free mass index Mean±SD	Fat mass Mean±SD	Fat mass index Mean±SD
*Body mass index*						
	<P 85	124	32.5±11.3 *	13.4±3.9 *	7.1±3.2 *	3.6±1.7 *
	>P 85	59	40.7±11.9 *	16.1±3.4 *	17.3±5.9 *	7.2±1.8 *

*Bipolar fat*						
	<25%	148	33.8±12.6 **	13.7±4.2 **	8.3±4.4 *	4.0±1.9 *
	>25%	31	38.9±10.2 **	16.1±3.2 **	19.6±6.0 *	8.4±1.5 *

*Waist circumference*						
	<P 85	138	32.1±12.0 *	13.3±4.3 *	7.4±3.7 *	3.9±1.9 *
	>P 85	45	44.0±8.8 *	16.9±1.4 *	19.1±4.8 *	7.5±1.9 *

*Systolic blood pressure*						
	<P 90	161	34.1±12.4 **	14.0±4.2	9.8±6.2 **	4.7±2.4
	>P 90	21	41.2±11.1 **	15.8±2.0	13.8±7.4 **	5.3±2.7

*Diastolic blood pressure*						
	<P 90	172	34.7±12.5	14.1±4.2	10.2±6.4	4.7±2.4
	>P 90	11	39.1±9.2	15.5±1.6	12.0±5.6	4.9±2.8

*Physical activity*						
	>300min/week	47	35.0±14.0	14.1±4.7	9.6±6.0	4.7±2.4
	<300min/week	134	34.9±11.9	14.2±3.9	10.5±6.6	4.8±2.5

*Sexual maturation*						
	Prepubertal	68	29.0±11.0*^a^	12.9±4.6 ** ^a^	9.2±5.9^a^	5.0±2.6^a^
	Pubertal	105	38.9±11.5*^b^	15.0±3.4 ** ^b^	11.0±6.5^a^	4.6±2.4^a^
	Postpubertal	4	35.1±24.3*^a^	12.2±8.3 ** ^a^	11.7±11.5^a^	6.2±3.1^a^

*Total cholesterol*						
	<150mg/dL	84	38.0±13.0 **	14.7±4.1	11.2±6.5	4.9±2.4
	>150mg/dL	92	33.5±10.0 **	14.2±3.4	9.9±6.1	4.6±2.4

*Glucose*						
	<100mg/dL	167	35.5±11.9	14.4±3.8	10.4±6.1 **	4.7±2.4
	>100mg/dL	6	40.5±9.3	16.0±2.1	17.0±9.8 **	6.5±3.5

*Triglycerides*						
	<100mg/dL	149	35.2±12.4	14.3±3.9	9.8±6.0 *	4.5±2.3 **
	>100mg/dL	27	38.0±7.0	15.4±1.8	14.6±7.1 *	5.9±2.9 **

*High density lipoprotein cholesterol*						
	>45mg/dL	76	31.9±12.4 *	13.3±4.4 *	8.5±5.1 *	4.4±2.2
	<45mg/dL	69	39.1±10.4 *	15.4±2.6 *	12.5±6.8 *	5.1±2.6

*Low density lipoprotein cholesterol*						
	<100mg/dL	99	36.0±13.2	14.2±4.2	10.2±6.1	4.6±2.3
	>100mg/dL	46	33.9±8.9	14.7±2.7	10.9±6.6	4.9±2.6

Means with different letters are significantly different according to the Least Significant Difference test (LSD).Student's *t* -test.

* *p* <0.01;

** *p* <0.05.

In serum lipid values and fasting plasma glucose comparison, FFM was found to be higher in male adolescents with total cholesterol under 150mg/dL. Those with impaired fasting glucose had high FM. Moreover, males with serum triglycerides above 100mg/dL were characterized as having higher values of FM and FMI. It was found that adolescents with low serum levels of HDL-c had higher averages of FFM, FFMI, and FM.

## Discussion

Obesity in adolescence is reaching increasing proportions and results in 21.5% of Brazilian adolescents with excess weight and obesity.^1^ Among the students of Juiz de Fora who participated in the study, the prevalence was 19.9% for overweight and 10.2% for obesity.

The results for body composition showed differences between the sexes. Female adolescents showed higher percentage of body fat and mass and FMI, similar to the study by Eissa et al.^4^ For both sexes, the highest average values of FFM, FFMI, FM and FMI were related to the anthropometric variables, which estimate total and central body fat (BMI, % fat, and waist circumference).

Body composition varies widely in adolescents; it depends on age, gender, ethnicity, height, and sexual maturation.^5^ During puberty, for both sexes, there is a weight increase in lean and fat tissue and bone mineral content,^20^ but the major growth and development conditioning of this stage of life results from sexual maturation, which contributes to dimorphism between girls and boys.^21^

In this study, FFMI and FFM were characterized by an increase in its values according to the pubertal stage, particularly in girls. In the sexual maturation process, height, lean muscle mass, and bone mineral content are directly proportional to the peak height velocity^20^ and, therefore, higher FFM and FFMI were found in postpubertal stage, similar to that reported by Miranda et al.^22^ The same was seen for FM and FMI in female adolescents, whose highest averages occurred in the postpubertal stage.

It is known that a certain storage of body fat is needed for the occurrence of pubertal growth spurt in both sexes and that the relative amount of fat in females increases progressively during adolescence.^23^ In the study by Miranda et al.,^22^ the highest accumulation was found in the prepubertal stage. However, higher body fat mass values in the postpubertal stage may be seen in adolescents who reach menarche at an early age and who need to reach a critical weight of at least 17% fat to start the growth spurt and reach menarche. There is also the influence of high blood concentrations of estradiol to stimulate greater lipogenesis.^24^ Furthermore, most adolescents in this study were in puberty stage, and the small sample size of the pre- and postpubescent stages may have contributed to the outcomes.

As for the boys, there was no significant difference in mean values of FM and FMI between pubertal stages, which corroborates the study by Borges et al.^25^ For boys, the largest contributor to increase BMI seems to be the increase in FFM.^5^ Most of our adolescents were in pubertal stage, which is characterized by height growth and increased lean mass.

According to Leccia et al.,^26^ sexual maturation seems to have an effect on blood pressure and acts primarily on body size. They found a strong correlation between height, body weight, and lean body mass with systolic blood pressure for both boys and girls and with diastolic blood pressure just for girls. In our study, higher diastolic blood pressure was found in girls, compared to males, and FFM was higher among boys who had high systolic blood pressure, probably because the increased muscle mass was greater in male adolescents. Weight gain and high body fat can significantly contribute to high blood pressure even in individuals classified as normotensive.^27^ Systolic blood pressure of adolescents in this study was associated with higher mean values of FMI and its corresponding mass for both sexes. According to Jessup and Harrell,^28^ systolic blood pressure increases in pubertal stage, regardless of age, particularly in girls. In this study, we found that most of the adolescents of both sexes were in the pubertal stage of maturation.

Literature shows that excess body fat can increase the risk of metabolic disorders, such as dyslipidemia, insulin resistance, and impaired glucose tolerance.^29^ The results of this study indicated that FM and FMI were higher for both boys and for girls with high serum triglycerides. The boys with impaired fasting glucose also had higher FM and FMI values.

However, FFM values were higher in adolescents with low levels of serum HDL-c of both sexes and increased total cholesterol in male adolescents. Changes in body composition and lipid profile during puberty may be influenced by reduced physical activity and change in eating habits and hormonal, which is common in adolescence.^28^ Although this study did not consider dietary and hormonal information, there was a significant difference in physical activity and sedentary activities between the sexes. Male adolescents spend more time doing physical exercise than girls. In the study by Schubert et al.,^29^ the increase in FFMI was associated with decreased HDL-c and high triglycerides. It is possible that the increase in FFM, followed by higher elevations of FM, may result in reduced concentrations of HDL-C and that the FFMI effect is masked by that of FMI. Pietrobelli et al.^30^ also found that the relationship of increased lean body mass and reduced serum levels of HDL-c suggest that the increase in serum triglycerides leads to the formation of particles smaller than HDL and an increase in HDL metabolism.

This study has some limitations. Although pressure measurements were standardized, they were performed in a single day, and the periods of time of physical activity were estimated by self-report of adolescents. However, they are methodologies considered more practical, feasible, and validated for cross-sectional studies, like the one performed.^19^ Still, the results of this study indicate that adolescents with anthropometric, clinical, and biochemical characteristics considered as risk for developing cardiovascular disease had higher FMI values. Regarding FFMI, the differences were not marked. Different methodologies for assessment of body composition and, consequently, cardiovascular risks to which adolescents are predisposed make health promotion and the prevention of future damage more effective.
