# Supplementary material for: Association between fat mass index and fat-free mass index values and cardiovascular risk in adolescents
Source: Rev Paul Pediatr. 2016 Jan-Mar;34(1):30–7. doi: 10.1016/j.rppede.2015.06.020 (PMC4795719; doi:10.1016/j.rppede.2015.06.020)
Supplement: Translation

# Translation: Associação entre índice de massa de gordura e índice de massa livre de gordura e risco cardiovascular em adolescentes

**Authors:** Patrícia Morais de Oliveira, Fabiana Almeida da Silva, Renata Maria Souza Oliveira, Larissa Loures Mendes, Michele Pereira, Ana Paula Carlos Cândido

**Affiliations:** Universidade Federal de Juiz de Fora (UFJF), Juiz de Fora, MG, Brasil

**Keywords:** Doenças cardiovasculares/fatores de risco, Adolescente/crescimento e desenvolvimento, Composição corporal, Gordura corporal

## Abstract

**Objetivo:**

Descrever a relação entre valores de índice de massa de gordura e índice de massa livre de gordura e fatores associados a risco cardiovascular em adolescentes de Juiz de Fora (MG).

**Métodos:**

Estudo transversal feito com 403 adolescentes de 10-14 anos, de escolas públicas e privadas. Avaliaram-se medidas antropométricas, clínicas, bioquímicas, autorrelato do tempo dedicado ao exercício físico, atividades sedentárias e estágio de maturação sexual.

**Resultados:**

Quanto ao estado nutricional, 66,5% dos adolescentes estavam eutróficos, 19,9% com sobrepeso e 10,2% obesos. Para ambos os sexos, o índice de massa de gordura foi maior nos adolescentes que estavam com triglicerídeos séricos, índice de massa corporal e circunferência da cintura elevados.

**Conclusões:**

Os adolescentes que tinham características antropométricas, clínicas e bioquímicas consideradas de risco para o desenvolvimento de doenças cardiovasculares apresentaram maiores valores do índice de massa de gordura. Metodologias diferenciadas para avaliação da composição corporal tornam a promoção da saúde e a prevenção de agravos futuros mais eficazes.

## Introdução

A prevalência de excesso de peso em adolescentes vem aumentando,^1^ associa-se com fatores de risco para doenças cardiovasculares na vida adulta e repercute em elevados gastos para saúde pública^1^. Atualmente, demonstra-se que a distribuição da gordura corporal exerce influência maior do que a massa corporal total na presença dos fatores de risco cardiovasculares.^2^^-^ 4 A capacidade de estimar ou quantificar os estoques de gordura é central para a prevenção e o tratamento de doenças relacionadas à obesidade.^2^ Portanto, são indispensáveis métodos mais precisos para verificar a adiposidade e fazer triagem adequada para intervenção rápida.^3^^-^ 6

O índice de massa corporal (IMC) tem sido o método antropométrico mais usado para diagnóstico de excesso de peso, mas sua capacidade preditiva para identificar jovens com gordura corporal elevada gera discussões no meio científico.^5^^,^ 6 Esse índice não permite a distinção da massa gorda e massa magra, não reflete as grandes mudanças na composição corporal que ocorrem nessa faixa etária e que são distintas entre os sexos.^5^ Portanto, seria mais viável fazer a distinção dos componentes corporais por meio de medidas mais precisas que considerem o percentual de gordura corporal.^2^

Van Itallie *et al*.^7^ propuseram o uso do índice de massa de gordura (IMG) e do índice de massa livre de gordura (IMLG) para uma avaliação antropométrica mais criteriosa, de acordo com os compartimentos corporais, por cálculo que considera a quantidade em quilos da massa de gordura e de massa livre de gordura obtidas por bioimpedância elétrica, com a vantagem de relacionar apenas um componente do peso corporal com a altura ao quadrado e a de serem expressos em unidades comuns ao IMC.^8^ Com o uso desses dois índices, torna-se possível julgar se o déficit ou excesso de peso corporal se deve seletivamente a uma mudança na massa livre de gordura, na massa de gordura ou a ambas.^8^ Podem ser identificadas quatro situações típicas: baixa IMLG e alta IMG, correspondente à obesidade; baixa IMLG e baixa IMG, correspondente à magreza; alta IMLG e baixa IMG, correspondente à hipertrofia muscular; e alta IMLG e alta IMG, correspondente a excesso de massa de gordura e massa livre de gordura combinadas. Ainda não são um consenso na literatura científica os valores de referência desses dois índices, principalmente para adolescentes. No estudo de Nakao e Komiya,^9^ os valores de referência de IMLG para meninos foram de 12,7-13,4kg/m^2^ e para as meninas de 12-13kg/m^2^. Já para o IMG, os valores de referência adotados foram de 2,8-3,6kg/m^2^ em meninos e 3,2-3,8kg/m^2^ em meninas.

Considerando a necessidade de avaliação antropométrica mais criteriosa em adolescentes, o objetivo deste trabalho foi descrever a relação entre valores de índice de massa de gordura e índice de massa livre de gordura e fatores associados a risco cardiovascular em adolescentes de Juiz de Fora (MG).

## Método

Trata-se de um estudo transversal feito com adolescentes de 10-14 anos, de escolas públicas e privadas de Juiz de Fora (MG). O número de escolas e de alunos por instituição, pertencentes à referida faixa etária, foi obtido por meio do Censo Escolar fornecido pelo Inep de 2009^10^. Foram selecionadas 35 escolas, com base na proporção por regiões da cidade. A amostra total levou em conta três parâmetros: proporção da população na faixa etária estudada com obesidade (8%)^11^; admitiu-se a precisão desejada de 2%, com nível de significância de 5%; e consideraram-se 20% de perdas devido às ausências das crianças nos dias das coletas de dados ou às recusas (não consentimento da criança ou dos pais/responsáveis). Os estudantes foram selecionados aleatoriamente por meio da tabela de números aleatórios e estratificados de acordo com sexo, idade e proporção em cada escola.

Avaliaram-se medidas antropométricas, clínicas e bioquímicas. Os estudantes ainda responderam quanto ao tempo gasto ao praticar atividade física, assistir televisão, jogar videogame e usar o computador. Os adolescentes que praticavam acima de 300 minutos/semana de atividade física foram considerados ativos.^12^ A escala de Tanner^13^ foi usada para identificação autoavaliativa do estágio de maturação sexual em que os adolescentes estavam e as meninas indicaram a presença e a idade em que ocorreu a menarca.

A massa corporal foi aferida em balança dotada de bioimpedância bipolar (Tanita^®^BC-553, Illinois, EUA) com capacidade máxima de 136kg, também usada para estimar o percentual de gordura corporal. Aferiu-se a estatura por meio de estadiômetro de campo (Alturaexata^*®*^, Belo Horizonte, Brasil), com escala em centímetros e precisão de 0,01m. O estado nutricional dos adolescentes foi determinado por meio do IMC por idade, segundo o critério de classificação estabelecido pela OMS, em que valores de IMC superiores ao percentil 85 são classificados como sobrepeso.^14^ A circunferência da cintura foi obtida por fita métrica simples e inelástica de 1,5m e intervalo de 0,01m. Todas as medições seguiram os procedimentos normatizados pelo OMS e valores de circunferência acima do percentil 85 foram considerados como elevados para ambos os sexos. Quanto ao percentual de gordura, valores superiores a 30% para meninas e 25% para meninos foram considerados elevados e de risco.^15^

Usou-se bioimpedância tetrapolar Biodynamics 310^®^ (Biodynamics Corporation, Washington, EUA), com as aferições em conformidade com as instruções do aparelho. Os valores de resistência e reactância encontrados foram usados para determinar a quantidade em quilogramas de massa de gordura (MG) e de massa livre de gordura (MLG). O IMG foi determinado pela divisão da massa de gordura (em quilogramas) pela altura ao quadrado (em metros). Da mesma forma, o IMLG foi calculado como a divisão da massa magra (em quilogramas) pelo quadrado da altura (em metros), conforme proposto por Van Itallie *et al*.^7^ Fez-se o somatório das médias dos dois valores mais próximos referentes às medidas das espessuras das dobras cutâneas biciptal, tricipital, subescapular e suprailíaca. As médias das dobras cutâneas tricipital e subescapular também foram somadas separadamente. O plicômetro analógico Lange^®^ (Beta Technology, Califórnia, EUA), com precisão de 0,1mm e em triplicata, foi empregado para aferir a dobra cutânea bicipital (feita com os cotovelos fletidos em 90°, meio caminho entre a axila e a fossa cubital), triciptal (no ponto médio da distância entre o acrômio e o olecrânio, na face posterior do braço esquerdo), subescapular (no ângulo inferior da escápula, em diagonal a 45°) e suprailíaca (sobre a linha média axilar, entre a última costela e a crista ilíaca). A fórmula de Deurenberg *et al*.^16^ foi usada para análise dos dados com intenção de predizer valores associados à densidade corporal e, posteriormente, aos de gordura em relação ao peso corporal.

As amostras de sangue (6mL) foram coletadas por punção venosa na região antecubital dos pacientes com 12 horas de jejum para dosagem de colesterol total e frações (HDL-c e LDL-c), triglicerídeos. Os procedimentos foram normatizados no Laboratório de Bioquímica do Hospital Universitário da Universidade Federal de Juiz de Fora (UFJF). Para avaliar as alterações lipídicas, os valores de referência preconizados pela Sociedade Brasileira de Cardiologia foram aplicados.^17^ Os valores acima de 150mg/dL, 100mg/dL e 100mg/dL para colesterol total, triglicerídeos e LDL-c, respectivamente, foram considerados elevados e de risco para adolescentes de ambos os sexos. Já para o HDL-c, considerou-se risco valores abaixo de 45mg/dL, tanto para rapazes quanto para moças. O diagnóstico de *diabetes mellitus* foi determinado por meio dos critérios estabelecidos pela Sociedade Brasileira de Diabetes (glicemia de jejum acima de 100mg/dL).^18^ Os parâmetros foram analisados pelo analisador automático Cobas Mira Plus^*®*^ (Roche Diagnostics, Suíça).

Os níveis de pressão arterial dos alunos submetidos à avaliação foram mensurados por três vezes com o aparelho oscilométrico digital Omron HEM-705CP^*®*^ (Omron Corporation, Brasil). Os indivíduos estavam sentados (braço esquerdo estendido na altura do coração) e respeitou-se um intervalo de 10-15 minutos entre cada aferição. A classificação foi baseada na V Diretriz Brasileira de Hipertensão Arterial a partir da média das três aferições feitas (pressão arterial sistólica e pressão arterial diastólica acima do percentil 90).^19^

O projeto foi aprovado pelo Comitê de Ética da Universidade Federal de Juiz de Fora, parecer n° 09/2010, e iniciado após o consentimento dos responsáveis legais dos adolescentes e diretores dos estabelecimentos de ensino.

Na análise estatística usou-se o teste *t* de Student para avaliar as diferenças entre os valores médios de cada variável em relação ao sexo. A análise de variância (Anova) foi usada para comparação de médias dos índices de composição corporal e suas massas correspondentes com as demais variáveis do estudo. O *software* SPSS versão 17.0 foi usado para as análises estatísticas e admitiu-se um nível de significância de 5%.

## Resultados

Foram avaliados 403 adolescentes, 218 (54,1%) do sexo feminino, com idade média de 12,4±1,2 anos. A distribuição da faixa etária foi semelhante entre os sexos (*p*=0,30). A amostra foi composta por 66,5% de adolescentes eutróficos, 19,9% com sobrepeso e 10,2% obesos. A tabela 1 mostra as características da população do estudo estratificada por sexo.

**Tabela 1:** Comparação de valores médios das variáveis demográficas, antropométricas, clínicas, bioquímicas e de atividade física da população em estudo, por sexo (n=403). Juiz de Fora, MG, Brasil, 2014

	Meninas (n=218)			Meninos (n=185)		
Variável	n	Média ± DP		n	Média ± DP	*p* ‐valor a
Idade (anos)	218	12,4±1,1		185	12,3±1,1	0,30
Altura (cm)	217	155,5±8,5		185	155,8±10,4	0,74
Peso (Kg)	217	49,3±13,2		185	48,0±12,8	0,33
IMC (Kg/m 2 )	218	20,2±4,6		185	19,5±3,5	0,16
Gordura corporal bipolar (%)	209	25,3±7,8		179	17,5±7,7	<0,01
Circunferência cintura (cm)	213	66,9±9,9		183	67,7±9,7	0,39
Somatório 4 dobras (mm)	197	60,7±26,1		170	50,1±26,0	<0,01
Somatório dobra tricipital e subescapular (mm)	197	31,5±13,1		170	26,7±13,1	<0,01
Massa Livre de Gordura (Kg)	218	33,9±8,3		185	35±12,4	0,30
Índice de Massa Livre de Gordura	218	13,9±2,9		185	14,2±4.1	0,42
Massa de gordura (Kg)	218	13,9±8,8		185	10,3±6,4	<0,01
Índice de Massa de Gordura	218	5,7±3,4		185	4,8±2,5	<0,01
PA sistólica média (mmHg)	218	109,0±9,9		184	110,3±10,8	0,22
PA diastólica média (mmHg)	218	68,1±6,5		184	66,5±7,0	<0,05
Tempo Atividade Física (min/sem)	213	171,4±120,8		181	269,1±165,7	<0,01
Tempo assistindo a TV, videogame e computador (horas/semana)	213	8,0±4,5		183	10,6±5,4	<0,01
Colesterol total (mg/dL)	202	153,1±26,2		176	150,4±25,9	0,32
Glicemia (mg/dL)	201	82,2±7,9		173	82,4±9,6	0,88
Triglicerídeos (mg/dL)	202	69,6±30,7		176	67,2±32,3	0,47
HDL‐c (mg/dL)	162	48,7±8,9		145	46,7±11,2	0,09
LDL‐c (mg/dL)	162	90,9±23,3		145	90,8±20,5	0,95

a Teste *t* de Student.

IMC, índice de massa corporal; PA, pressão arterial; HDL‐c, *High Density Lipoproteins* ; LDL‐c, *Low Density Lipoproteins* .

Em relação à composição corporal, as adolescentes apresentaram maiores percentuais de gordura corporal (determinada por bioimpedância bipolar e somatório das dobras cutâneas), MG e maior IMG. A pressão arterial diastólica média foi maior no sexo feminino. Os adolescentes do sexo masculino gastaram mais tempo na prática de atividade física e também em práticas sedentárias, como assistir a TV, jogar videogame e usar o computador, do que as adolescentes.

As tabelas 2 e 3 representam o valor médio e desvio padrão de características antropométricas, clínicas e bioquímicas em relação aos índices de composição corporal e suas massas correspondentes para adolescentes do sexo feminino e masculino, respectivamente. Para ambos os sexos, valores médios superiores de MLG, IMLG, MG e IMG foram encontrados em adolescentes com o excesso de peso, gordura corporal e circunferência da cintura elevadas. Houve diferença significativa na pressão arterial sistólica. As meninas com PAS >p90 apresentaram valores médios maiores de MG e IMG e os meninos, na mesma classificação de PAS, valores médios maiores de MG e MLG. Em relação à maturação sexual, o IMLG e a MLG encontravam-se mais elevados no estágio pós-púbere, tanto para meninas quanto para os meninos. O mesmo foi observado para a MG e o IMG nas adolescentes no estágio pós-púbere. Na avaliação bioquímica, as adolescentes com HDL-c<45mg/dL apresentaram maiores médias de MLG, IMLG, MG e IMG. Já as dosagens de triglicerídeos foram superiores nas adolescentes com MG e IMG elevados.

**Tabela 2:** Comparação entre as características antropométricas, clínicas, bioquímicas e de atividade física em relação à MLG, IMLG, MG e IMG de adolescentes do sexo feminino. Juiz de Fora, MG, Brasil, 2014

Variáveis		n	MLG	IMLG	MG	IMG
			Média ± DP	Média ± DP	Média ± DP	Média ± DP
*IMC*						
	<P85	155	32,1±7,3 *	13,4±2,6 *	10,0±4,4 *	4,1±1,6 *
	>P85	62	38,7±7,7 *	15,5±2,4 *	24,0±8,9 *	9,6±3,3^a^ *

*Gordura bipolar*						
	<30%	154	32,2±7,3 *	13,4±2,6 *	10,2±4,4 *	4,2±1,7 *
	>30%	54	39,2±8,0 *	15,6±2,6 *	25,0±9,0 *	9,9±3,4 *

*Circunf. Cintura*						
	<P85	165	32,3±7,2 *	13,5±2,5 *	10,6±4,8 *	4,4±1,9 *
	>P85	48	39,3±8,2 *	15,5±2,8 *	25,3±9,6 *	10,0±3,6 *

*PA sistólica*						
	<P90	198	33,7±7,6	13,9±2,5	13,2±7,7 *	5,4±2,99 *
	>P90	19	35,7±13,9	13,9±5,2	21,8±13,9 *	8,5±5,4 *

*PA diastólica*						
	<P90	202	34,1±7,8	14,0±2,6	13,9±8,2	5,6±3,1
	>P90	15	31,1±13,3	12,9±5,3	15,1±14,4	6,2±5,7

*Atividade física*						
	>300min/semana	21	35,0±6,3	14,8±1,6	15,2±9,4	6,3±3,6
	<300min/semana	192	33,7±8,5	13,8±3,0	13,8±8,7	5,6±3,3

*Maturação sexual*						
	Pré‐pubere	54	28,6±8,1*^a^	13,0±3,3*^a^	9,5±6,5* ^a^	4,2±2,7*^a^
	Púbere	150	35,4±6,7*^b^	14,2±2,4*^b^	14,8±7,6*^b^	5,9±3,0*^b^
	Pós‐pubere	7	43,9±7,4*^c^	16,2±1,9*^b^	31,7±17,2*^c^	11,8±6,7*^c^

*Colesterol total*						
	<150mg/dL	94	34,8±8,9	13,9±3,2	14,0±8,1	5,6±3,1
	>150mg/dL	108	33,5±8,0	13,9±2,7	14,4±9,4	5,8±3,6

*Glicemia*						
	<100mg/dL	198	34,0±8,5	13,9±3,0	14,1±8,8	5,7±3,4
	>100mg/dL	3	34,1±4,5	13,7±0,8	14,8±10,1	5,8±3,9

*Triglicerídeos*						
	<100mg/dL	171	33,7±8,6	13,8±3,1	13,5±8,0 **	5,4±3,0 **
	>100mg/dL	31	36,2±7,2	14,8±1,8	17,7±12,1 **	7,2±4,7 **

*HDL‐c*						
	>45mg/dL	111	32,7±8,4 *	13,4±3,1 *	12,9±8,1 *	5,2±3,1 *
	<45mg/dL	51	37,0±8,3 *	14,8±2,8 *	18,1±10,0 *	7,2±3,9 *

*LDL‐c*						
	<100mg/dL	104	34,4±9,2	13,8±3,4	14,2±8,0	5,6±3,0
	>100mg/dL	58	33,4±7,3	14,1±2,4	15,3±10,7	6,3±4,1

MLG, massa livre de gordura; IMLG, índice de massa livre de gordura; MG, massa de gordura; IMG, índice de massa de gordura; IMC, índice de massa gorporal; Circunf. cintura, circunferência da cintura; PA, pressão arterial; HDL‐c, High Density Lipoproteins; LDL‐c, Low Density Lipoproteins. Médias com letras diferentes apresentam diferenças significativas de acordo com o teste Least Significant Difference (LSD).Teste *t* de Student

* *p* <0,01;

** *p* <0,05.

**Tabela 3:** Comparação entre as características antropométricas, clínicas, bioquímicas e de atividade física em relação à MLG, IMLG, MG e IMG de adolescentes do sexo masculino. Juiz de Fora, MG, Brasil, 2014

Variáveis		n	MLG	IMLG	MG	IMG
			Média ± DP	Média ± DP	Média ± DP	Média ± DP
*IMC*						
	<P85	124	32,5±11,3 *	13,4±3,9 *	7,1±3,2 *	3,6±1,7 *
	>P85	59	40,7±11,9 *	16,1±3,4 *	17,3±5,9 *	7,2±1,8 *

*Gordura bipolar*						
	<25%	148	33,8±12,6 **	13,7±4,2 **	8,3±4,4 *	4,0±1,9 *
	>25%	31	38,9±10,2 **	16,1±3,2 **	19,6±6,0 *	8,4±1,5 *

*Circunferência da cintura*						
	<P85	138	32,1±12,0 *	13,3±4,3 *	7,4±3,7 *	3,9±1,9 *
	>P85	45	44,0±8,8 *	16,9±1,4 *	19,1±4,8 *	7,5±1,9 *

*PA sistólica*						
	<P90	161	34,1±12,4 **	14,0±4,2	9,8±6,2 **	4,7±2,4
	>P90	21	41,2±11,1 **	15,8±2,0	13,8±7,4 **	5,3±2,7

*PA diastólica*						
	<P90	172	34,7±12,5	14,1±4,2	10,2±6,4	4,7±2,4
	>P90	11	39,1±9,2	15,5±1,6	12,0±5,6	4,9±2,8

*Atividade física*						
	>300min/semana	47	35,0±14,0	14,1±4,7	9,6±6,0	4,7±2,4
	<300min/semana	134	34,9±11,9	14,2±3,9	10,5±6,6	4,8±2,5

*Maturação sexual*						
	Pré‐púbere	68	29,0±11,0*^a^	12,9±4,6 ** ^a^	9,2±5,9^a^	5,0±2,6^a^
	Púbere	105	38,9±11,5*^b^	15,0±3,4 ** ^b^	11,0±6,5^a^	4,6±2,4^a^
	Pós‐pubere	4	35,1±24,3*^a^	12,2±8,3 ** ^a^	11,7±11,5^a^	6,2±3,1^a^

*Colesterol total*						
	<150mg/dL	84	38,0±13,0 **	14,7±4,1	11,2±6,5	4,9±2,4
	>150mg/dL	92	33,5±10,0 **	14,2±3,4	9,9±6,1	4,6±2,4

*Glicemia*						
	<100mg/dL	167	35,5±11,9	14,4±3,8	10,4±6,1 **	4,7±2,4
	>100mg/dL	6	40,5±9,3	16,0±2,1	17,0±9,8 **	6,5±3,5

*Triglicerídeos*						
	<100mg/dL	149	35,2±12,4	14,3±3,9	9,8±6,0 *	4,5±2,3 **
	>100mg/dL	27	38,0±7,0	15,4±1,8	14,6±7,1 *	5,9±2,9 **

*HDL‐c*						
	>45mg/dL	76	31,9±12,4 *	13,3±4,4 *	8,5±5,1 *	4,4±2,2
	<45mg/dL	69	39,1±10,4 *	15,4±2,6 *	12,5±6,8 *	5,1±2,6

*LDL‐c*						
	<100mg/dL	99	36,0±13,2	14,2±4,2	10,2±6,1	4,6±2,3
	>100mg/dL	46	33,9±8,9	14,7±2,7	10,9±6,6	4,9±2,6

MLG, massa livre de gordura; IMLG, índice de massa livre de gordura; MG, massa de gordura; IMG, índice de massa de gordura; IMC, índice de massa corporal; Circunf. cintura, circunferência da cintura; PA, pressão arterial; HDL‐c, High Density Lipoproteins; LDL‐c, Low Density Lipoproteins. Médias com letras diferentes apresentam diferenças significativas de acordo com o teste Least Significant Difference (LSD).Teste *t* de Student

* *p* <0,01;

** *p* <0,05.

Na comparação com os valores de lipídeos séricos e glicemia de jejum, observou-se que a MLG foi maior nos rapazes com colesterol total menor do que 150mg/dL. Aqueles que estavam com glicemia de jejum alterada apresentaram MG elevada. Além disso, os rapazes com triglicerídeos séricos acima de 100mg/dL caracterizaram-se por contar com maiores valores de MG e IMG. Observou-se que adolescentes com níveis séricos reduzidos de HDL-c apresentaram maiores médias de MLG, IMLG e MG.

## Discussão

A obesidade na adolescência vem atingindo proporções cada vez maiores e resulta em 21,5% de adolescentes brasileiros com sobrepeso e obesidade.^1^ Entre os escolares de Juiz de Fora deste estudo, as prevalências foram de 19,9% de sobrepeso e 10,2% de obesidade.

Os resultados referentes à composição corporal foram diferentes entre os sexos. As adolescentes mostraram maior massa e percentual de gordura corporal e IMG, similar ao estudo de Eissa *et al*.^4^ Para ambos os sexos, os maiores valores médios de MLG, IMLG, MG e IMG relacionaram-se às variáveis antropométricas, que estimam gordura corporal total e central (IMC, % gordura e circunferência da cintura).

A composição corporal varia muito em adolescentes, depende de idade, sexo, etnia, altura e maturação sexual.^5^ Durante a puberdade, para ambos os sexos, há um aumento de peso em tecido magro, tecido adiposo e conteúdo mineral ósseo,^20^ porém o maior condicionamento do crescimento e desenvolvimento dessa fase é dado pela maturação sexual, que contribui para o dimorfismo entre meninas e meninos.^21^

Na presente investigação, o IMLG e a MLG foram caracterizadas por aumento em seus valores de acordo com o estágio puberal, especialmente nas meninas. No processo de maturação sexual, a estatura, a massa muscular magra e o conteúdo mineral ósseo são diretamente proporcionais ao pico de velocidade de crescimento em estatura^20^ e, por isso, foi encontrado maior MLG e IMLG no estágio pós-púbere, similar ao referido por Miranda *et al*.^22^ O mesmo observou-se para a MG e o IMG nas adolescentes, em que as maiores médias ocorreram no estágio pós-púbere.

Sabe-se que é preciso um determinado depósito de gordura corporal para que ocorra o estirão puberal para ambos os sexos e que a quantidade relativa de gordura no sexo feminino aumente progressivamente durante a adolescência.^23^ No estudo de Miranda *et al*.,^22^ o maior acúmulo foi verificado na fase pré-púbere. Entretanto, valores de massa de gordura corporal superiores no estágio pós-púbere podem ser verificados em adolescentes que atingem a menarca em idade precoce e que necessitam alcançar um peso crítico constituído por, no mínimo, 17% de gordura para iniciar o estirão de crescimento e atingir a menarca. Há também a influência das concentrações sanguíneas elevadas de estradiol que estimulam maior lipogênese.^24^ Além disso, a maioria das adolescentes do presente estudo estava no estágio púbere e o pequeno tamanho amostral dos estágios pré e pós-púbere pode ter contribuído para os resultados encontrados.

Já para os rapazes, não houve diferença significativa nos valores médios de MG e IMG entre os estágios puberais, o que corrobora o estudo de Borges *et* al.^25^ Para os meninos, o que mais contribui para o aumento do IMC parece ser o aumento da MLG.^5^ A fase púbere, em que a maioria dos adolescentes do estudo estava, caracteriza-se pelo crescimento da altura e aumento da massa magra.

Conforme Leccia et al.,^26^ a maturação sexual parece exercer efeito sobre a pressão arterial e age principalmente no tamanho corporal. Os autores encontraram forte correlação entre a altura, o peso corporal e a massa corporal magra com a pressão arterial sistólica tanto para meninos quanto para meninas e para a pressão arterial diastólica, apenas para meninas. Em nosso estudo verificou-se maior pressão arterial diastólica em meninas, comparadas com o sexo masculino, e a MLG foi maior nos rapazes que apresentaram pressão arterial sistólica elevada, provavelmente porque o aumento da massa muscular foi mais acentuado nos adolescentes do sexo masculino. O ganho de peso e a adiposidade corporal elevada podem contribuir significativamente para a elevação da pressão sanguínea mesmo em indivíduos classificados como normotensos.^27^ A pressão arterial sistólica dos adolescentes deste estudo associou-se com valores médios maiores do IMG e sua massa correspondente para ambos os sexos. Segundo Jessup *et al*.,^28^ a pressão arterial sistólica aumenta na fase púbere, independentemente da idade, particularmente em meninas. No presente trabalho, notou-se que a maior parte dos adolescentes de ambos os sexos estava na fase púbere de maturação.

A literatura mostra que o excesso de gordura corporal pode aumentar os riscos de alterações metabólicas, como dislipidemias, resistência à insulina e tolerância diminuída à glicose.^29^ Os resultados deste estudo indicaram que a MG e o IMG foram superiores tanto para meninos quanto para meninas com níveis séricos elevados de triglicerídeos. Os rapazes que apresentaram glicemia de jejum alterada também foram os que tiveram maiores valores de MG e IMG.

Porém, observaram-se maiores valores de MLG em adolescentes com níveis séricos reduzidos de HDL-c para ambos os sexos e aumentados de colesterol total para os meninos. As alterações na composição corporal e no perfil lipídico durante a puberdade podem ser influenciadas pela reduzida atividade física e mudança nos hábitos alimentares e hormonais, comuns na adolescência.^28^ Embora este trabalho não tenha considerado informações dietéticas e hormonais, verificou-se diferença significativa quanto à prática de atividade física e de atividades sedentárias entre os sexos. Os adolescentes do sexo masculino gastaram mais tempo com a prática de exercícios físicos do que as meninas. No estudo de Schubert *et al*.,^29^ o aumento do IMLG associou-se à diminuição de HDL-c e ao aumento de triglicerídeos. É possível que o aumento da MLG, acompanhado por maiores elevações da MG, possa resultar em concentrações reduzidas de HDL-c e que o efeito do IMLG seja mascarado pelo do IMG. Pietrobelli *et al*.^30^ também encontraram essa relação do aumento da massa magra corporal e redução dos níveis séricos de HDL-c e sugerem que o aumento de triglicerídeos séricos conduz à formação de menores partículas de HDL e ao aumento do catabolismo de HDL.

Algumas limitações do presente estudo devem ser consideradas. Apesar das aferições de pressão terem sido feitas de forma padronizada, elas foram feitas em um único dia e período e o tempo de atividade física foi estimado pelo autorrelato dos adolescentes. Entretanto, são metodologias consideradas mais práticas, viáveis e validadas para estudos transversais, como o que foi feito.^19^ Ainda assim, os resultados deste estudo indicam que adolescentes com características antropométricas, clínicas e bioquímicas consideradas de risco para o desenvolvimento de doenças cardiovasculares apresentaram maiores valores de IMG. Em relação ao IMLG, as diferenças não foram acentuadas. Metodologias diferenciadas para avaliação da composição corporal e, consequentemente, dos riscos cardiovasculares aos quais os adolescentes estão predispostos tornam a promoção da saúde e a prevenção de agravos futuros mais eficazes.
